# Two novel e13a3 variants detected in CML patients and their DNA breakpoint analysis

**DOI:** 10.3389/fonc.2026.1932890

**Published:** 2026-08-12

**Authors:** Dong Liang, Jianmei Chang, Zhifang Xu, Fanggang Ren, Binai Yang, Xiuhua Chen, Hongwei Wang

**Affiliations:** 1 Department of Blood Transfusion, Peking University First Hospital Taiyuan Hospital (Taiyuan Central Hospital), Taiyuan, China; 2 The Key Laboratory of Molecular Diagnosis and Treatment of Hematological Diseases of Shanxi Province, the Second Hospital Of Shanxi Medical University, Taiyuan, China

**Keywords:** aberrant splicing, BCR::ABL1, chronic myelogenous leukemia, DNA breakpoint, e13a3

## Abstract

Even in the era of tyrosine kinase inhibitor (TKI) therapy, the prognostic landscape of atypical transcripts in chronic myeloid leukemia (CML) remains poorly defined. In our preliminary work, we identified two atypical transcripts harboring distinct DNA breakpoints that ultimately translate into an identical truncated protein. To investigate the DNA breakpoints of the *BCR::ABL* fusion gene in two patients with novel e13a3-type CML and to clarify the underlying mechanism of their generation, we conducted a breakpoint analysis. Target DNA fragments were amplified via nested PCR using specific primers, followed by agarose gel purification and sequencing analysis. RT-PCR sequencing identified two distinct e13a3 transcript variants: an 819-bp insertion was detected between BCR exon 13 and ABL exon 3 in patient 1, whereas a 131-bp insertion was found in patient 2. DNA sequencing verified that the breakpoint regions were located in BCR intron 13 and ABL intron 2, which were fully consistent with the cDNA fusion sites. The intronic sequence insertion induced a frameshift, leading to identical premature translation termination at BCR intron 13 in both cases. This resulted in the complete deletion of the entire ABL region, generating a truncated protein with 20-amino acids at the C-terminus of BCR exon 13. This novel e13a3 transcript originates from DNA breakage. Both patients achieved remission after imatinib treatment; molecular docking simulation results show that e13a3 has a lower binding energy with imatinib than e13a2. This finding suggests that in the TKI era, more attention should be paid to the types of truncated proteins resulting from DNA breakpoints.

## Introduction

The Philadelphia (Ph) chromosome originates from the reciprocal translocation t(9; 22)(q34;q11), which generates the *BCR::ABL1* fusion gene. This fusion is detected in 95% of patients with chronic myeloid leukemia (CML) and in about 20% of adult patients with acute lymphoblastic leukemia (ALL) (1, 2). On chromosome 22, the *BCR* gene contains three different breakpoint cluster regions (M-bcr, m-bcr, and μ-bcr), whereas the breakpoints on chromosome 9 consistently occur within intron 1 of the (*ABL proto-oncogene 1, ABL1*) gene. As a result, typical *BCR* breakpoints fuse to *ABL1* exon 2, producing *BCR*-a2-type transcripts such as e13a2, e14a2, or e1a2 (3). By contrast, *BCR*-a3-type transcripts, which occur between the *BCR* gene and *ABL* exon 3, are uncommon. To date, about 96 cases carrying *BCR*-a3-type transcripts have been reported (2, 4–31), including 33 cases (**Table 1**) with e13a3. To the best of our knowledge, all previously described cases involved typical transcripts characterized by the direct fusion of a *BCR* exon with an *ABL1* exon at the complementary DNA (cDNA) level. In this paper, we first describe for the first time two novel atypical *BCR*-a3-type transcripts, which contain inserted intronic sequences from both the *BCR* and *ABL1* genes at the fusion site. Two hypotheses may account for these observations: (i) aberrant splicing results in fused intronic sequences that are not completely but only partially spliced; (ii) although both *BCR* and *ABL1* genes generate breaks and rearrangements in the genomic DNA intronic region, the fused intronic sequences may be fully recognized as a pseudo-exon and therefore not spliced as an intron sequence. To address this issue, we developed a “*breakpoint analysis*” for two patients with *BCR*-a3-type transcripts.

**Table 1:** Statistics of reported atypical transcript e13a3 in CML.

Refs. (subtypes)	e13a3 counts	Age	Sex	Karotype	Formation mechanism	Splicing type	Treatment	Outcome	Follow-up	Diagnosis stage	Country
(9)	3 (2,231, 0.1%)	/	/	Ph+	Aberrant splicing	e13a3	Imatinib	CCyR, MMR	1 year	CML-CP	China
(11)	1	25	Female	Complex karyotype	Aberrant splicing	e13a3	/	/		CML-CP	Germany
(16)	6	37 (median)	/	Ph+	Aberrant splicing	e13a3	Imatinib	CCyR, MMR	12 months	CML-CP	China
(17)	4	/	/	Ph+	Aberrant splicing	e13a3	/	3 for CCyR and MMR; 1 for CCyR	2 years	CML-CP	Germany
(18)	2	46 (median)	/	Ph+	Aberrant splicing	e13a3	Imatinib	/	/	/	China
(23)	5	/	Male	Ph+	Aberrant splicing	e13a3	/	/	/	/	China
(19)	1	24	Female	ph(−); FISH(+)	Aberrant splicing	e13a3	Imatinib	CCyR, MMR	2 years	CML-CP	China
(20)	1	32	Male	Ph+	Aberrant splicing	e13a3	Hydroxyurea + nilotinib	CCyR, MMR	/	CML-CP	China
(21)	1	89	Male	Ph+	Aberrant splicing	e13a3	Hydroxyurea + imatinib + dasatinib	CCyR, MMR	5 years	CML-CP	Italy
(25)	1	39	Male	Ph+	Aberrant splicing	e13a3	Nilotinib	CCyR, MMR	4 years	CML-CP	China
(26)	1	66	Male	Ph+	Aberrant splicing	e13a3	Imatinib	MMR	14 months	CML-CP	Ireland
(27)	1	49	Male	Ph+	DNA breakpoint	e13a3	IFN-a + hydroxyurea + imatinib	CCyR, MMR	20 months	CML-CP	Japan
(28)	1	30	Male	Ph+	Aberrant splicing	e13a3	Ponatinib + FLAG-IDA + transplantation	CCyR, MMR	19 months	CML-CP	UK
(29)	4	33.5 (median)	3 males; 1 female	Ph+	Aberrant splicing	e13a3	Imatinib	3 for CCyR and MMR; 1 for CCyR	About 2~6 years	3 for CML-CP; 1 for CML-BC	India
(31)	1	57	Male	Ph+	Aberrant splicing	e13a3	Nilotinib	CCyR, MMR	18 months	CML-CP	Korea

CHR, complete hematologic remission; MMR, major molecular response; CCyR, complete cytogenetic response; CML-CP, chronic myeloid leukemia-chronic phase; CML-BC, chronic myeloid leukemia-blast crisis.

/, not acquired.

## Materials and methods

### Patients

Two patients with *BCR*-a3-type-positive CML were identified at the Secondary Hospital of Shanxi Medical University. The diagnosis was established according to the World Health Organization Classification of Neoplastic Diseases of the Hematopoietic and Lymphoid Tissues. Written informed consent was obtained from each patient. Bone marrow smears were analyzed in both cases, and clinical data were extracted from medical records at the time of bone marrow or peripheral blood collection.

### Nested PCR and sequencing

The genomic DNA was extracted from the cells of bone marrow smears using Chelex 100 reagent (Sigma-Aldrich Corp., St. Louis, MO, USA). Taking into account the lower concentration of the isolated DNA, we further applied nested polymerase chain reaction (nested PCR) to perform analysis with specific primers. The primer sequences were as follows: F: 5′ cagactgtccacagcattccg3′; R_1_: 5′ggcccatggtaccaggagtgtttctccag3′; R_2_: 5′ggcgtgatgtagttgcttggg3′; R_3_: 5′gattatagcctaagacccggagc3′. All patients were tested using the same primers. The first PCR was performed with primers F and R_1_, the second with primers F and R_2_, and the third with primers F and R_3_. PCR reaction was performed in a 25-µl reaction volume with 20 µM of forward primer or reverse primer, 2.5 µl of 10× Taq buffer, 1.5 µl of 25 mM MgCl_2_, 0.5 µl of 10 mM dNTPs, and 1.5 U of Taq DNA polymerase (Fermentas, Glen Burnie, MD, USA). PCR amplification was performed as follows: 94 °C for 5 min, 30 cycles of 94 °C for 30 s, 60 °C for 30 s, and 72 °C for 30 s, followed by 10 min at 72 °C. The PCR products were separated by 2% agarose gels and purified using the Gel Extraction Kit (Takara Bio Inc., Otsu, Shiga, Japan). Sequencing analyses were performed by Beijing Huada Biotechnology Company (Beijing Huada Gene Technology Co., Ltd., Daxing District, Beijing, China) and analyzed by GenBank BLAST.

### Molecular docking

Molecular docking simulations were performed using AutoDock Vina version 1.2.5 to compare the binding profiles of imatinib to the protein products encoded by the atypical e13a3 transcript and the canonical e13a2 transcript.

All included subjects signed an informed consent form. The study protocol complied with the ethical principles of the Declaration of Helsinki and was approved by the ethics committee of the Second Hospital of Shanxi Medical University.

## Result

**Figure 1:**
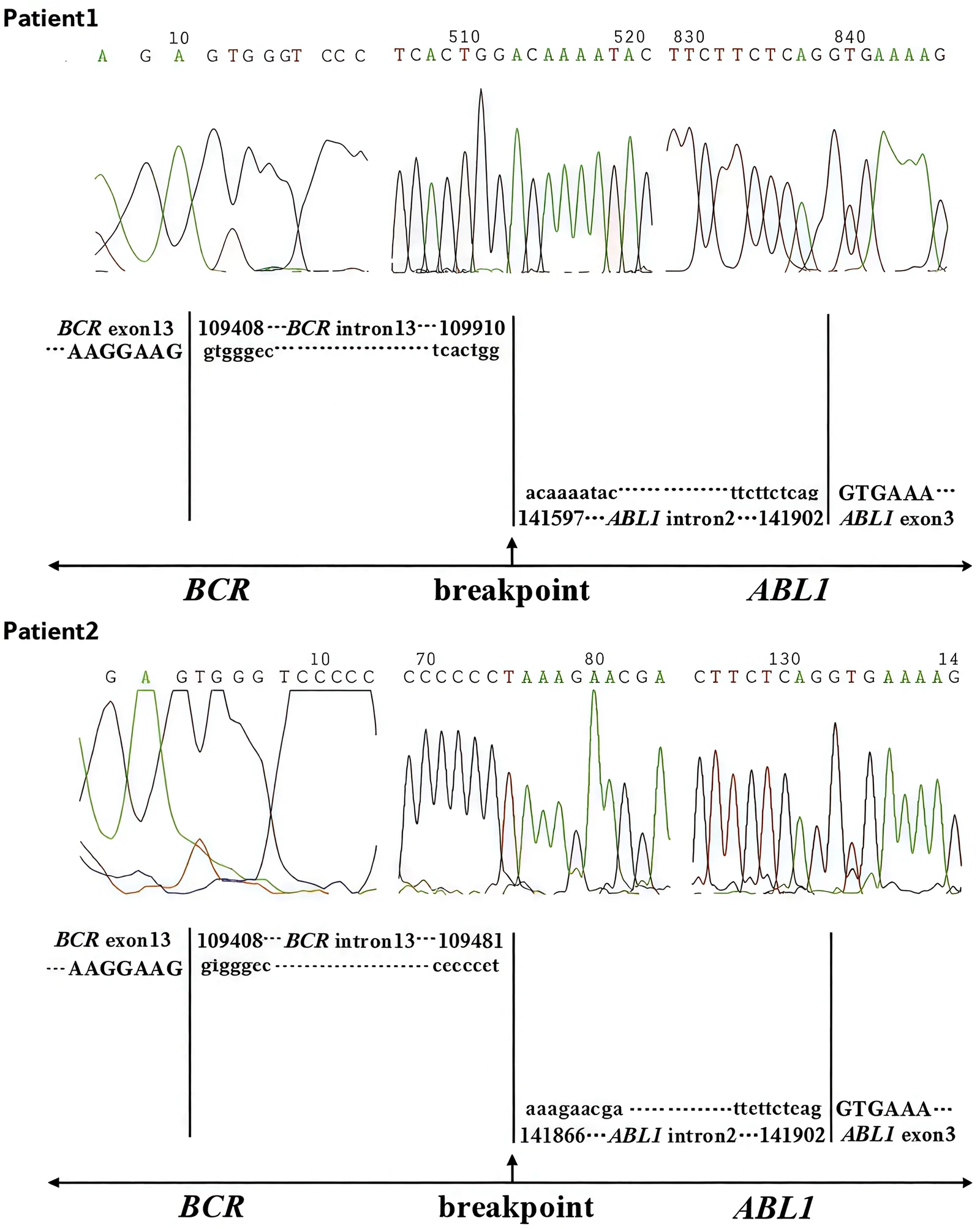
DNA sequence of two novel e13a3 transcripts and a hypothetical breakpoint model of the BCR and ABL1 sequences during the formation of the e13a3 fusion transcript. In BCR, the break occurred in intron 13, downstream of exon 13, whereas in ABL1, the break site was found in intron 2, upstream of exon 3.

**Figure 2:**
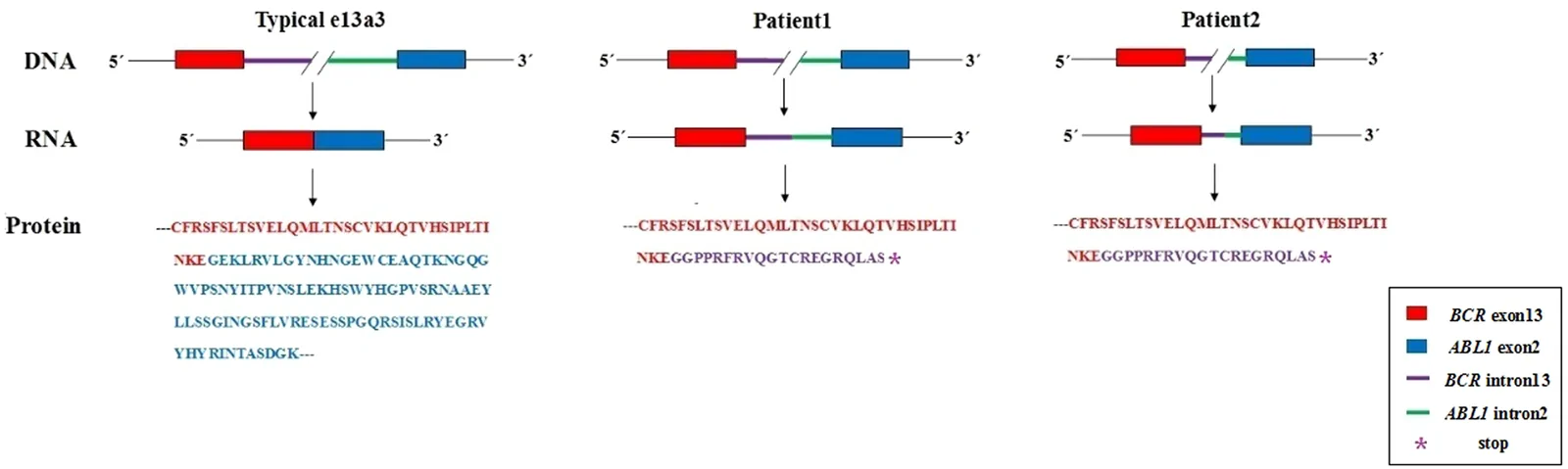
Schema chart of the generation of the two variant e13a3 transcripts.

Patient 1 was a 64-year-old woman diagnosed with CML. Initial bone marrow BCR::ABL1 mRNA sequence analysis revealed an atypical e13a3 transcript with inserted intronic sequences, which were formed by the joining of a 503-bp nucleotide sequence from the 5′ part of *BCR* intron 13 (nt 109408–109910, NC_000022) and a 326-bp nucleotide sequence from the 3′ part of ABL1 intron 2 (nt 141597–141902, NC_000009). Furthermore, genomic DNA analysis showed that the breakpoints occurred 503 bp downstream of the *BCR* exon 13 and 326 bp upstream of the *ABL* exon 2 (**Figure 1**), suggesting that the fused intronic sequence was not completely spliced at the cDNA level. It is worth mentioning that the 829-bp inserted intronic sequence between *BCR* exon 13 and *ABL1* exon 3 led to a frameshift of 20-amino acids followed by a stop codon and produced a truncated BCR::ABL1 protein (**Figure 2**). Subsequently, treatment with imatinib mesylate was started, and 6 months later, this BCR::ABL1 chimeric transcript was no longer detectable.

Patient 2 was a 30-year-old man diagnosed with CML. Initial molecular analysis by reverse transcription polymerase chain reaction (RT-PCR) revealed only a common e13a2-type *B*CR::ABL1 transcript. After the patient had been treated with imatinib mesylate for 12 months, the BCR::ABL1 transcript could still be detected. However, the RT-PCR product was not consistent with the previous e13a2 fusion type by size after electrophoresis. Further sequence analysis revealed an atypical e13a3-type transcript with inserted intronic sequences, which was a fusion of a 74-bp nucleotide sequence from the 5′ part of *BCR* intron 13 (nt 109408–109481, NC_000022) and a 57-bp nucleotide sequence from the 3′ part of *ABL1* intron 2 (nt 141866–141902, NC_000009). In order to explore its possible formation mechanism, we analyzed the genomic DNA breakpoints of the atypical e13a3-type transcript. The result showed that its breakpoints were located 74 bp downstream of the *BCR* exon 13 and 57 bp upstream of the *ABL1* exon 2 (**Figure 1**), suggesting that the fused intronic sequence was not completely spliced as an intron sequence at the cDNA level like patient 1. Interestingly, the 131 bp insertion disrupted the reading frame between *BCR* and *ABL1* and produced a premature stop codon, which was the same as patient 1 and encoded an identical truncated oncoprotein (**Figure 2**). The atypical transcript disappeared when the patient continued to be treated with imatinib mesylate for 6 months.

### Molecular docking simulation of imatinib and e13a3/e13a2 binding

**Figure 3:**
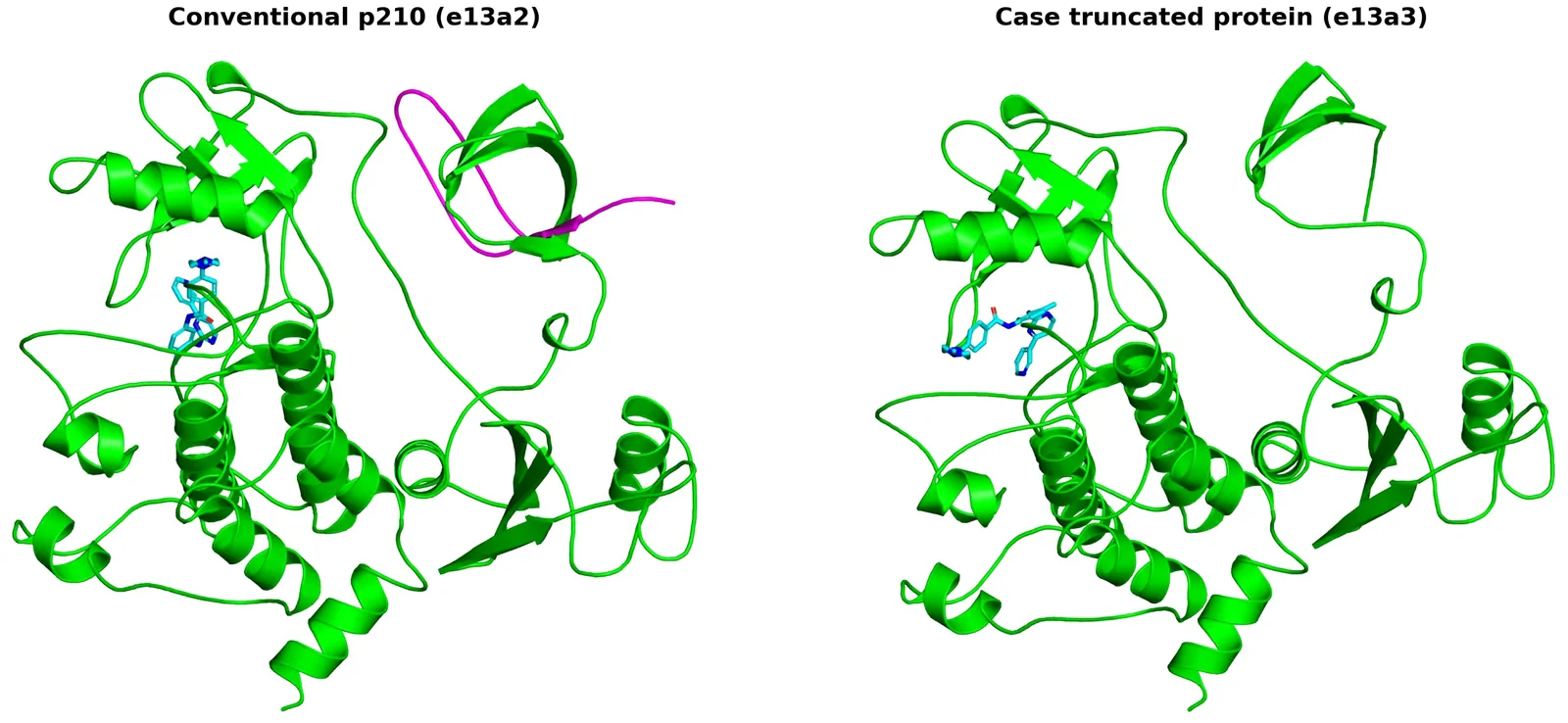
Molecular docking simulation of imatinib binding to the conventional transcript e13a2 and truncated transcript e13a3.

The optimal Vina binding energy between the canonical e13a2 transcript and imatinib is − 7.189 kcal/mol, while the binding energy between the e13a3 truncated protein reported in this study and imatinib is − 7.227 kcal/mol, with a difference of − 0.038 kcal/mol. The truncated protein reported in this study exhibits a lower binding energy with imatinib. **Figure 3** shows the molecular dynamics simulation diagrams of the canonical e13a2 transcript and the truncated e13a3 transcript bound to imatinib, where the pink region represents the deleted segment of the truncated protein.

## Discussion

In CML, besides the canonical transcripts e13a2 (b2a2) and e14a2 (b3a2), the atypical transcripts currently reported include e1a2, e1a3, e6a2, e8a2, e12a2, e13a3, e14a3, e16a2, and e19a2. Among these, the most common atypical transcripts are e13a3 and e14a3. This study aggregated all published cases of chronic myeloid leukemia harboring the rare atypical e13a3 transcript, yielding a total of 33 cases (**Table 1**) with a predominance of male patients.

Among these cases, 10 lacked information on their therapeutic regimens. Of the remaining 23 cases, 16 achieved favorable prognoses after imatinib monotherapy (9, 16, 18, 19, 26, 29); one patient failed to attain hematologic remission following 1 year of treatment with interferon combined with hydroxyurea and achieved molecular remission 8 months after switching to imatinib (27); one patient received sequential transplantation after imatinib therapy and maintained sustained remission at 19 months posttransplant. The remaining cases treated with second-generation tyrosine kinase inhibitors (TKIs) also obtained favorable prognoses (28). Both patients reported in this study achieved remission after 1 year of imatinib treatment, which is fully consistent with the therapeutic outcome characteristics reported in the published literature. Only one out of 33 cases had the e13a3 transcript derived from DNA breakage, with the others resulting from alternative splicing.

Here is the report on two rare BCR-a3-type Philadelphia-positive CML cases, which share structural transcripts containing different intronic sequences from the *BCR* and *ABL1* genes, yet produce the same truncated protein. Furthermore, in this study, we verified that the retained introns in both cDNA sequences were consistent with their own genomic DNA, indicating that the inserted introns in cDNA sequences resulted from DNA rearrangement rather than aberrant splicing. The data support the second hypothesis mentioned above. However, another question remains unclear. It is well-known that splicing events strictly comply with the “gt … ag” consensus rule in humans. The fused intronic sequences of the two atypical cases contain complete intronic splice sites (gt … ag), but they were not recognized and spliced as intron sequences at the cDNA level. Similarly, Sadia et al. also described an atypical e13a2 transcript with an analogous structure in the literature (15). To our knowledge, it is unusual for a variant transcript with a breakpoint occurring within both *BCR* and *ABL1* introns to result from the fusion of intronic sequences with complete splice sites from both *BCR* and *ABL1* genes. We speculated that it may be associated with the loss of some specific conserved intron sequences, which may be important in the intronic splicing process.

Usually, the BCR::ABL1 TKI imatinib mesylate has an inhibitory effect on *ABL1* kinase activity of the BCR::ABL1 oncoprotein. Based on the predictive analysis of protein structure, unexpectedly, we found that both patient 1 and patient 2 expressed an identical truncated oncoprotein of the *BCR::ABL1* gene, which lacks the *ABL1* kinase region and retains BCR exon 13, with an additional 20 intron-encoded amino acids followed by the C-terminus, leading to a frameshift and early termination.

Molecular docking simulation of the canonical e13a2 transcript and the e13a3 truncated transcript reported in this study showed that the latter has a slightly lower binding energy with imatinib. This simulation conclusion is fully consistent with the clinical findings in the published literature indicating that patients with atypical e13a3 transcripts can achieve remission rates comparable to or even faster than those with canonical e13a2 transcripts after TKI therapy. Meanwhile, the two particular cases responded quickly and completely to imatinib. Molecular simulation indicates that this phenomenon arises from oncoprotein conformational change, which modulates ABL1 kinase-like activity to drive myeloid proliferation and induce imatinib response.

Although the previously reported atypical transcripts e13a3 have been shown to achieve rapid remission with TKI inhibitor therapy, their overall proportion among CML cases remains low compared with that of typical transcripts, with frequencies of approximately 0.3%~4.6% as reported by Wang’s group and 1.7% as reported by Qin’s group (15, 16). The two atypical transcripts described in this study differ from all previously reported cases in that they result from gene breakage and produce the same truncated protein. The team led by Leske was the first to confirm the primary asciminib resistance mechanism of the e13a3 transcript via cellular experiments, and this finding indicates that drug selection is particularly critical for patients with atypical transcripts. These two cases highlight an important point: when evaluating atypical transcripts, it is essential to determine whether the transcripts are truly identical. This report expands the known diversity of e13a3 variants and lays the groundwork for future multicenter investigations.

## Data Availability

The raw data supporting the conclusions of this article will be made available by the authors, without undue reservation.
